# Efficacy and safety of floating needle therapy in the treatment of knee osteoarthritis: A protocol for systematic review and meta-analysis

**DOI:** 10.1097/MD.0000000000024505

**Published:** 2021-02-12

**Authors:** Qun Wan, Gen Deng, Sushun Yang, Ying Jiang, Qilin Zhao

**Affiliations:** a College of Acupuncture and Massage, Jiangxi University of Traditional Chinese Medicine; b Affiliated Hospital of Jiangxi University of Traditional Chinese Medicine, Nanchang, China.

**Keywords:** floating needle therapy, knee osteoarthritis, protocol, systematic review and meta-analysis

## Abstract

**Background:**

Knee osteoarthritis (KOA) is a common clinical degenerative disease of the joints, which is prone to occur in middle-aged and elderly people. Its early manifestations include knee redness, swelling, pain, effusion, sound, and so on. With the development of the disease, KOA will also lead to joint deformity and disability, which will seriously affect the living ability of patients. It not only brings physical pain and dysfunction to patients, but also leads to anxiety, helplessness, depression, and social disorder in social psychology, which seriously affects patients’ daily life, social function and life quality, and also brings huge economic burden and pressure to family and social medical treatment. Floating needle therapy has shown strong advantages in the treatment of KOA, and the curative effect is accurate. Therefore, this paper will carry out a systematic evaluation and meta analysis of the efficacy and safety of floating needle therapy in the treatment of KOA.

**Methods:**

We will search 8 electronic databases, including PubMed, Embase, Web of Science, Cochrane Library, the China National Knowledge Infrastructure, Chinese Science and Technology Periodical Database, Wanfang Database, and Chinese Biomedical Literature Database. We will search above electronic databases from the beginning to January 2021, without any language restriction. Clinical efficacy, including total effective rate or cure rate, visual analogue scale pain score, and recurrence rate will be accepted as the primary outcomes. The changes of traditional Chinese medicine symptom score, inflammatory factor level change, knee function score will be used as secondary outcomes. RevMan 5.3 software will be used for statistical analysis. The result about the curative effect and safety of floating needle therapy for knee osteoarthritis will be presented as risk ratio for dichotomous data and mean differences with a 95% confidence interval for continuous data.

**Results:**

Only when we finish this meta analysis can we get the result, no results are available at this timed.

**Conclusions:**

The results of this study will provide reliable evidence for the efficacy and safety of floating needle therapy in the treatment of knee osteoarthritis.

**INPLASY Registration number:**

INPLASY2020120145.

## 1 Introduction

Knee osteoarthritis (KOA), also known as degenerative osteoarthritis of the knee, is the most common chronic, progressive, and degenerative joint disease after middle age (over 40 years old). It is characterized by degeneration of articular cartilage, osteosclerosis, and hyperplasia. The main clinical manifestations are progressive knee pain, swelling, stiffness, dysfunction, and in severe cases, joint deformity, or even loss of joint function, affecting the normal life and work of patients.^[1]^ KOA is the main cause of lower extremity pain and mobility disorder in the elderly. Although it does not endanger the patient life like tumor, morbidity and high rheumatic or rheumatoid arthritis, ankylosing spondylitis, but due to the prevalence is higher, the result of joint discomfort that dysfunction of the quality of life of patients and the influence of social public health can not be ignored, not only bring patients physical pain, dysfunction, also caused the social psychological anxiety, helplessness, depression, social disorder, and so on, the serious influence the patient's daily life, social function and quality of life, at the same time for the family and community health are of great economic burden and pressure.^[2]^ At present, KOA is mostly treated with drugs to relieve symptoms, improve joint function, and protect cartilage, such as glucosamine, anti-inflammatory and analgesic drugs (oral and intra-articular injection), and end-stage surgical treatment such as artificial joint replacement, but the efficacy is not lasting and the recurrence rate is high.^[3]^ With the popularization and application of floating needle therapy in clinical practice, more and more literatures have been reported on the clinical efficacy of floating needle therapy in the treatment of KOA, and a large number of literatures have shown that floating needle therapy has a good effect in the treatment of KOA.^[4,5]^ However, there is still a lack of systematic evaluation on the efficacy and safety of floating needle therapy for KOA in clinical practice. Therefore, the effectiveness and safety of floating needle therapy in the treatment of KOA will be systematically evaluated and meta-analyzed in this paper.

## 2 Methods

### 2.1 Study registration

This protocol was registered with the International Platform of Registered Systematic Review and Meta-Analysis Protocols (INPLASY) on December 30, 2020 and was last updated on December 30, 2020 (registration number INPLASY2020120145.).

### 2.2 Inclusion criteria for study selection

#### 2.2.1 Types of studies

Clinical randomized controlled trials (RCTs) containing floating needle therapy for KOA will be included, with no limitation of language and publication status.

#### 2.2.2 Types of participants

There are clear and recognized diagnostic criteria and efficacy criteria, and all patients are diagnosed as KOA, regardless of gender, age, and origin of the case.

#### 2.2.3 Types of interventions

##### 2.2.3.1 Experimental interventions

Floating needle therapy includes all combination therapies using any type of floating needle therapy or based on floating needle therapy in combination with other therapies.

##### 2.2.3.2 Control interventions

The control group will receive one of the following treatment methods: conventional pharmacological therapy, no treatment, and placebo.

#### 2.2.4 Types of outcome measures

##### 2.2.4.1 Primary outcome

Clinical efficacy, including total effective rate or cure rate, visual analogue scale pain score, and recurrence rate will be accepted as the primary outcomes.

##### 2.2.4.2 Secondary outcomes

The changes of traditional Chinese medicine symptom score, inflammatory factor level change, knee function score will be used as secondary outcomes.

### 2.3 Exclusion criteria

Non-RCTs; no exact diagnostic scale or therapeutic scale; no floating needle therapy as the main treatment in the experimental group, and floating needle therapy was found in the control group. Repeated literature; theory and review literature; animal experiments; nursing research.

### 2.4 The retrieval methods and strategies of this study

#### 2.4.1 Electronic database retrieval

We will search 8 electronic databases, including PubMed, Embase, Web of Science, Cochrane Library, the China National Knowledge Infrastructure, Chinese Science and Technology Periodical Database, Wanfang Database, and Chinese Biomedical Literature Database. We will search above electronic databases from the beginning to January 2021, without any language restriction. And will searching the relevant literature by combining subject words with free words, search terms consist of disease (“knee osteoarthritis” or “Knee joint osteoarthritis” or “KOA” or “Osteoarthritis of knee joint” or “knee pain” or “gonalgia” or “gonyalgia” or “Gonatalgia”) and intervention (“Floating needle” or “floating needle therapy” or “Fu's Subcutaneous Needling”) and research types (“randomized controlled trial” or “controlled clinical trial” or “random trials” or “RCT” or “RCTS”). The PubMed search strategy is shown in Table 1.

**Table 1:** Retrieval strategies in PubMed.

ID	Query
#1	“knee osteoarthritis”[Mesh]
#2	((((((Knee joint osteoarthritis[Ti/Ab]) OR (Osteoarthritis of knee joint[Ti/Ab])) OR (knee pain[Ti/Ab])) OR (KOA[Ti/Ab])) OR (gonalgia[Ti/Ab])) OR (gonyalgia[Ti/Ab])) OR (Gonatalgia[Ti/Ab])
#3	#1 OR #2
#4	“Floating needle”[Mesh]
#5	(floating needle therapy[Ti/Ab]) OR (Fu's Subcutaneous Needling[Ti/Ab])
#6	#4 OR #5
#7	(((randomized controlled trial[Ti/Ab]) OR (random trials[Ti/Ab])) OR (controlled clinical trial[Ti/Ab])) OR (RCT[Ti/Ab])
#8	#3 AND #6 AND #7

Ab = abstract, Mesh = medical subject headings, Ti = title.

#### 2.4.2 Searching other resources

We will combine manual retrieval of literature resource database to search relevant conference papers that meet the inclusion criteria. In addition, the grey literature, as well as ongoing and recently completed studies, will be searched on Clinicaltrials.gov.

### 2.5 Data extraction and management

#### 2.5.1 Literature inclusion and data extraction

**Figure 1:**
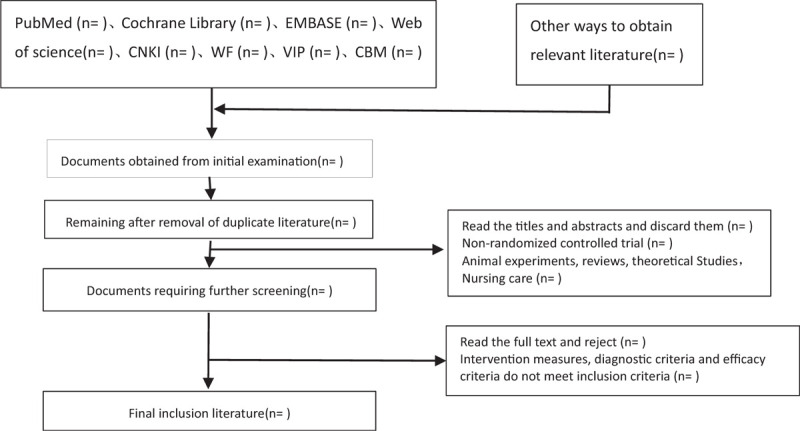
Literature screening flow chart.

The 2 researchers independently read the title and abstract of the literature we obtained, read the full text of the trials that might meet the inclusion criteria to determine whether the inclusion criteria were truly met, and discussed the conflicting literatures or let the third researcher decide whether to include them. Two researchers independently extracted data from the included studies, including study design, intervention measures and methods, measurement indicators, results, methodological contents such as hidden grouping and blind method, and so on, and a third evaluator checked the consistency of the data. If the required information is incomplete, we will contact the original author for the required data. The inclusion process of this study will be carried out as shown in Figure 1.

#### 2.5.2 Methodological quality evaluation

Two evaluators independently select the literature according to the inclusion and exclusion criteria and cross-check. In case of disagreement, a third evaluator will assist in the decision. The extracted data included the first author, year of publication, number of patients, age, gender, intervention measures, outcome indicators, and so on. The Jadad scale to evaluate quality into literature, including: random sequence (right 2 points, 1 points not clear, inappropriate 0), distribution, hidden (right 2 points, 1 points not clear, inappropriate 0), blinded (right 2 points, 1 points not clear, inappropriate 0), lost to follow-up and exit (describe 1 points, not describe 0); 0 to 3 is classified as low quality and 4 to 7 as high quality.

### 2.6 Statistical analysis

#### 2.6.1 Quantitative data synthesis

Meta analysis will be performed using Rev Man5.3.0 software. The odds ratio (OR) and its 95% confidence interval will be used as the counting data, while the weighted mean difference and its 95% confidence interval will be used as the measurement data.

#### 2.6.2 Assessment of heterogeneity

The heterogeneity test will be carried out first among all studies, *I*^2^ test will be used. When *P* > .1 and *I*^2^ < 50%, the fixed effect model will be used; otherwise, the random effect model will be used. When the clinical heterogeneity between the 2 studies is large, only descriptive analysis will be performed.

#### 2.6.3 Publication bias

When the number of qualified RCTs is sufficient, we will use the inverted funnel Egger to test the potential publication bias.

#### 2.6.4 Subgroup analysis

If heterogeneity exists in the meta-analysis, the source of heterogeneity should be sought, such as whether the degree of disease, treatment cycle, treatment time of each floating needle, type of intervention, and so on, is the source of heterogeneity. If so, a subgroup analysis should be conducted for these reasons to see whether heterogeneity still exists after analysis.

#### 2.6.5 Sensitivity analysis

Sensitivity analysis can not only assess the stability and reliability of the combined results, but also assess whether the combined results are significantly changed by the influence of a single study. If sufficient literature is included, we will adopt the method of excluding literature one by one, excluding each included study one by one before effect-size combination, changing the inclusion and exclusion criteria or excluding certain types of literature before effect-size combination.

## 3 Discussion

KOA is a common clinical degenerative disease of the joints, which is prone to occur in middle-aged and elderly people. Its early manifestations include knee redness, swelling, pain, effusion, sound, and so on. With the development of the disease, KOA will also lead to joint deformity and disability, which will seriously affect the living ability of patients.^[6]^ Western medicine treats KOA mainly by analgesia, anti-inflammatory, artificial joint replacement, and other methods. Although certain clinical effects have been achieved, the adverse reactions are relatively large.^[7]^ KOA is called “knee bi” in traditional Chinese medicine, its pathogenesis is due to wind, cold, wet, heat, and other evil gas blocking channels and collaterals, affecting blood and gas operation, resulting in limb bones, joints, muscles and other places of pain heavy, sour and numbness, or joint flexion and extension adverse, stiff, swollen, deformation, and other conditions.^[8]^ A large number of clinical reports have found that floating needle therapy for KOA can significantly reduce knee pain, stiffness, swelling and other symptoms, improve knee function and mobility, with good efficacy, quick effect, short course of treatment, and high safety.^[9]^ Floating needle therapy through the subcutaneous tissue around the local pain and scattered operation, loose connective tissue, have the effect of analgesia, the therapy not only retain the traditional acupuncture analgesia advantages of durable, safe, nontoxic side effects, and overcome the number of traditional acupuncture therapy, acupuncture, easy cause curved needle, needle breakage, hysteresis and shortcomings, and the operation method is simple and less time-consuming, patients are easy to accept.^[5,10]^ Therefore, it is necessary to systematically evaluate the treatment of KOA by floating needle therapy in this study, which can provide evidence-based medicine evidence for future clinical guidance of the treatment of KOA by floating needle therapy.

## Author contributions

**Data curation:** Qun Wan, Gen Deng.

**Formal analysis:** Qun Wan, Ying Jiang.

**Investigation:** Qun Wan, Gen Deng.

**Methodology:** Gen Deng, Ying Jiang.

**Project administration:** Sushun Yang, Qi Lin Zhao.

**Software:** Ying Jiang.

**Supervision:** Qun Wan, Qi Lin Zhao.

**Validation:** Sushun Yang, Qi Lin Zhao.

**Visualization:** Ying Jiang, Sushun Yang.

**Writing – original draft:** Qun Wan, Qi Lin Zhao.

**Writing – review & editing:** Qun Wan, Qi Lin Zhao.

## Abbreviations

Abbreviations: KOA = knee osteoarthritis, RCTs = randomized controlled trials.

## References

[1] Lu Y Shi X. Current situation and progress of domestic and international epidemiological research on knee osteoarthritis. Chin J Orthop Traumatol Tradit Chin Med 2012;20:81–4.

[2] Lu S Zhang R. Advances in the epidemiology of knee osteoarthritis in middle-aged and elderly people. Chin J Gerontol 2016;36:4133–5.

[3] Wu Y Zhang F Wu G. Clinical observation of exercise therapy combined with glucosamine hydrochloride tablets in the treatment of senile knee osteoarthritis. J Zhejiang Univ Chin Med 2020;44:1226–9.

[4] Zhu J. Clinical observation of floating needle therapy in the treatment of knee osteoarthritis. Guangming Chin Med 2019;34:3166–8.

[5] Chen Z Tan W He Z. Therapeutic effect of floating needle combined with four knee needles on knee osteoarthritis. Clin Study Tradit Chin Med 2017;9:104–5.

[6] Xie Y Ou H Chen X. Treatment of knee osteoarthritis by floating needle combined with warm acupuncture. Chin Med Med 2020;31:985–7.

[7] Kang L Wei Q. Advances in clinical research on the treatment of knee osteoarthritis with floating needle. J Gansu Univ Tradit Chin Med 2020;37:115–8.

[8] Xin L Zhang L Feng Q. Observation on the effect of acupuncture combined with fumigation of traditional Chinese medicine on knee osteoarthritis. Nurs Rehabil 2020;19:89–91.

[9] Before H Zhou S. Clinical study on the treatment of knee osteoarthritis with floating needle. J Pract Chin Med 2018;34:100–1.

[10] Huang Z Chen H Huang L. Effect comparison of floating needle therapy and electroacupuncture therapy in the treatment of knee osteoarthritis. Inner Mongolia Chin Med 2020;39:104–5.

